# Sarcopenia on preoperative chest computed tomography predicts cancer‐specific and all‐cause mortality following pneumonectomy for lung cancer: A multicenter analysis

**DOI:** 10.1002/cam4.4207

**Published:** 2021-08-19

**Authors:** Fabian M. Troschel, Qianna Jin, Florian Eichhorn, Thomas Muley, Till D. Best, Konstantin S. Leppelmann, Chi‐Fu Jeffrey Yang, Amelie S. Troschel, Hauke Winter, Claus P. Heußel, Henning A. Gaissert, Florian J. Fintelmann

**Affiliations:** ^1^ Department of Radiation Oncology Münster University Hospital Münster Germany; ^2^ Department of Radiology Division of Thoracic Imaging and Intervention Massachusetts General Hospital Boston Massachusetts USA; ^3^ Department of Diagnostic and Interventional Radiology with Nuclear Medicine Thoraxklinik Heidelberg at Heidelberg University Hospital Heidelberg Germany; ^4^ Department of Diagnostic and Interventional Radiology University Hospital Heidelberg Heidelberg Germany; ^5^ Translational Lung Research Centre (TLRC) Heidelberg German Centre for Lung Research Heidelberg Germany; ^6^ Department of Radiology Union Hospital Tongji Medical College Huazhong University of Science and Technology Wuhan China; ^7^ Department of Surgery Thoraxklinik, Heidelberg University Hospital Heidelberg Germany; ^8^ Department of Radiology Charité‐Universitätsmedizin Berlin Corporate Member of Freie Universität Berlin Humboldt‐Universität zu Berlin Berlin Institute of Health Berlin Germany; ^9^ Department of Surgery Division of Thoracic Surgery Massachusetts General Hospital Boston Massachusetts USA

**Keywords:** non‐small cell lung cancer, sarcopenia, survival

## Abstract

**Background:**

Mortality risk prediction in patients undergoing pneumonectomy for non‐small cell lung cancer (NSCLC) remains imperfect. Here, we aimed to assess whether sarcopenia on routine chest computed tomography (CT) independently predicts worse cancer‐specific (CSS) and overall survival (OS) following pneumonectomy for NSCLC.

**Methods:**

We included consecutive adults undergoing standard or carinal pneumonectomy for NSCLC at Massachusetts General Hospital and Heidelberg University from 2010 to 2018. We measured muscle cross‐sectional area (CSA) on CT at thoracic vertebral levels T8, T10, and T12 within 90 days prior to surgery. Sarcopenia was defined as T10 muscle CSA less than two standard deviations below the mean in healthy controls. We adjusted time‐to‐event analyses for age, body mass index, Charlson Comorbidity Index, forced expiratory volume in 1 second in % predicted, induction therapy, sex, smoking status, tumor stage, side of pneumonectomy, and institution.

**Results:**

Three hundred and sixty‐seven patients (67.4% male, median age 62 years, 16.9% early‐stage) underwent predominantly standard pneumonectomy (89.6%) for stage IIIA NSCLC (45.5%) and squamous cell histology (58%). Sarcopenia was present in 104 of 367 patients (28.3%). Ninety‐day all‐cause mortality was 7.1% (26/367). After a median follow‐up of 20.5 months (IQR, 9.2–46.9), 183 of 367 patients (49.9%) had died. One hundred and thirty‐three (72.7%) of these deaths were due to lung cancer. Sarcopenia was associated with shorter CSS (HR 1.7, *p* = 0.008) and OS (HR 1.7, *p* = 0.003).

**Conclusions:**

This transatlantic multicenter study confirms that sarcopenia on preoperative chest CT is an independent risk factor for CSS and OS following pneumonectomy for NSCLC.

## Graphical abstract

This transatlantic bi‐institutional study confirms sarcopenia on preoperative chest computed tomography (CT) as an independent binary risk factor for both cancer‐specific and overall survival following pneumonectomy for lung cancer. In the future, muscle mass on chest CT scans obtained as part of guideline‐concordant preoperative evaluation could be harnessed to improve mortality risk prediction in this patient cohort.

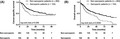

**Lay summary**

Removal of an entire lung (pneumonectomy) is a surgical procedure with high mortality risk. Thus, it is crucial to provide each patient with an individualized pre‐operative risk assessment. This transatlantic, bi‐institutional cohort study aimed to assess whether low muscle (sarcopenia) on chest computed tomography (CT) scans obtained for routine preoperative evaluation can identify patients with lung cancer at increased risk of death following pneumonectomy. Among 367 patients, we found that sarcopenia is an independent risk factor for death and that consideration of sarcopenia in addition to established predictors significantly improves the accuracy of cancer‐specific and overall survival estimation.

## 1 INTRODUCTION

Pneumonectomy in patients with non‐small cell lung cancer (NSCLC) is associated with high postoperative and long‐term mortality with reported averages of 5%–12% at 90 days^1^, ^2^, ^3^ and 60%–70% at 5 years.^1^, ^4^ Important known risk factors for mortality following pneumonectomy include clinical tumor stage,^5^ preoperative pulmonary function testing,^6^ comorbidities,^2^ and male sex.^5^ However, mortality risk prediction remains imperfect.^1^, ^7^, ^8^

Muscle cross‐sectional area (CSA) on CT scans obtained for routine care is a surrogate for muscle mass and therefore may diagnose sarcopenia.^9^, ^10^ Current evidence supporting low muscle mass as a predictor of early death after lung resection is predominantly based on single‐center cohorts of patients who underwent lobectomy, a less mortal, and morbid procedure compared to pneumonectomy.^11^ An association of OS after pneumonectomy with psoas muscle mass at the level of the third lumbar vertebra (L3) and thoracic muscle mass has been reported in two single‐center cohorts of about 150 patients each.^12^, ^13^ However, sarcopenia on chest CT based on externally validated cut‐off values and the link with cancer‐specific survival (CSS) have not been previously investigated in the context of pneumonectomy for NSCLC.

To confirm whether preoperative sarcopenia on chest CT predicts mortality following pneumonectomy in patients with lung cancer, we developed this bi‐institutional transatlantic cohort study in January 2019 with the a priori hypothesis that sarcopenia on preoperative chest CT is an independent risk factor for cancer‐specific and overall survival following pneumonectomy for NSCLC.

## 2 METHODS

This retrospective study was approved by the Institutional Review Boards at Massachusetts General Hospital (MGH; 2018P000301) and Heidelberg University Thoraxklinik (HDB; S‐295/2020). The need for informed consent was waived.

### 2.1 Inclusion and exclusion criteria

Consecutive adult patients undergoing standard and carinal pneumonectomy for non‐small cell lung cancer at the two institutions between January 1, 2010, and December 31, 2018, were included. Completion pneumonectomy, pleuropneumonectomy, and pneumonectomy with palliative intent were excluded.

**FIGURE 1:**
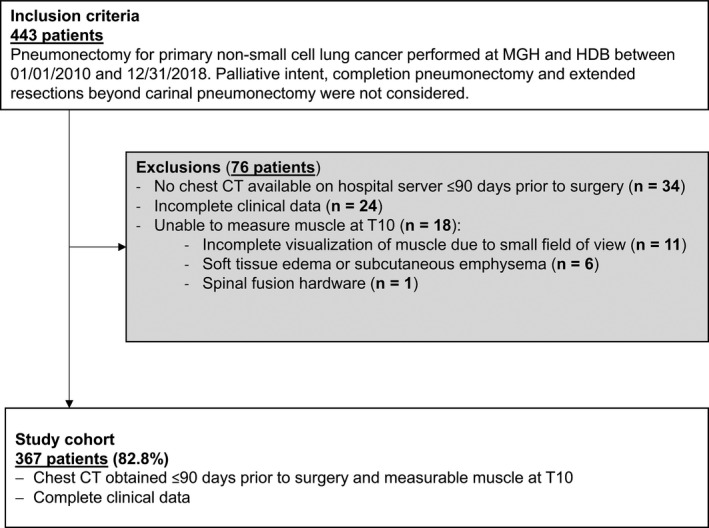
Study inclusion and exclusion criteria. MGH, Massachusetts General Hospital; HDB, Heidelberg University Thoraxklinik; CT, computed tomography; T10, level of the 10th thoracic vertebral body

Patients were identified using prospectively maintained registries, the institutional sample of the Society of Thoracic Surgeons (STS) General Thoracic Surgery Database (GTSD) at the MGH and the institutional cancer registry at HDB. Three exclusion criteria were applied (Figure 1): Patients without available chest CT scan within 90 days prior to pneumonectomy,^14^, ^15^ artifacts precluding assessment of sarcopenia at the T10 thoracic vertebral body,^16^ and incomplete clinical data.

### 2.2 Muscle measurements

**FIGURE 2:**
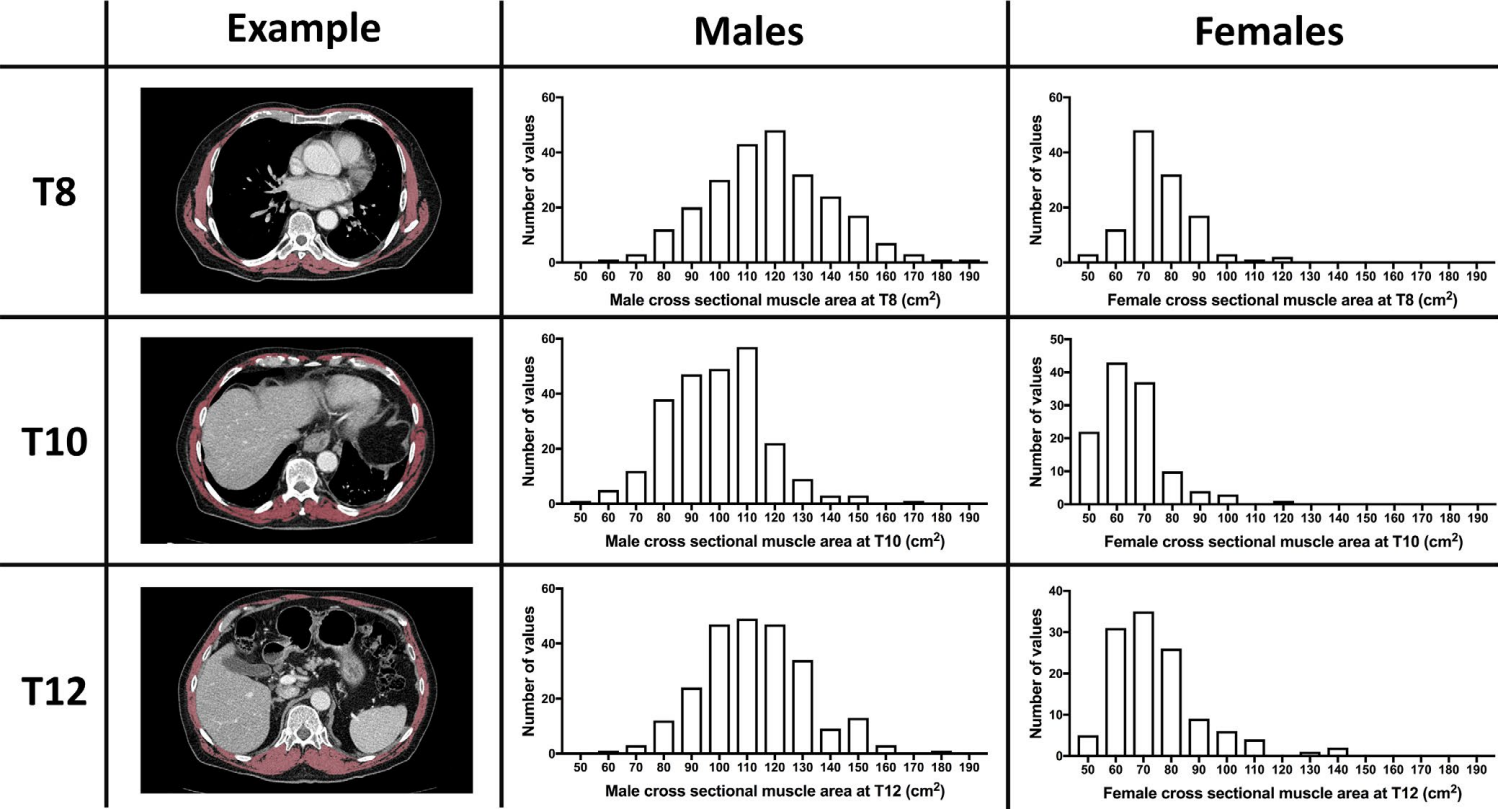
Representative example of muscle segmentation at three thoracic vertebral levels (left column) and distribution of cross‐sectional muscle area of all patients (males, middle column; females, right column). Left column: Pixels identified as muscle (red) superimposed on axial computed tomography images at the level of the 8th (T8), 10th (T10), and 12th (T12) thoracic vertebral body in a 79‐year‐old male. Histograms with muscle cross‐sectional area (in cm²) at each level for males (middle column) and females (right column)

Trained analysts (one radiation oncologist, one radiologist) blinded to outcomes independently quantified total muscle CSA on an axial image at the level of the T8, T10, and T12 thoracic vertebral bodies under the supervision of board‐certified thoracic radiologists (F.J.F., C.P.H.). Analysts used semi‐automated threshold‐based segmentation (−29 to +150 Hounsfield units) as illustrated in Figure 2 and previously described.^14^ Analysts used OsiriX Lite software (version 11.0.3, Pixmeo SARL, Bernex, Switzerland) at MGH and the Syngo Volume tool (Siemens Healthineers) at HDB. Twenty‐five randomly selected subjects were assessed with both software packages to determine inter‐software agreement. Two additional sets of 25 randomly selected subjects (one set per institution) were compared with the same software to determine inter‐analyst agreement.

### 2.3 Data collection, outcomes, and explanatory variable

Clinical data abstracted from prospective registries included patient demographics, co‐morbidities, tumor, and treatment details, as well as postoperative adverse events. Induction therapy was defined as pre‐operative chemotherapy, radiotherapy, or chemo‐radiotherapy. Pathologic tumor stage was uniformly classified according to the eighth edition of the tumor, node, and metastasis (TNM) classification for lung cancer.

The primary outcome was cancer‐specific survival and the secondary outcome was overall survival. Vital status and date of the last follow‐up were determined using the institutional registry, electronic medical record, and online obituaries, as previously described.^17^ Survival time was calculated in months from the date of resection. Patients without recorded death were censored at last contact as of 12/31/2019. The cause of death was categorized as *cancer*‐*specific*, *non*‐*cancer*‐*specific* or *unknown* by each institutional registry.

The explanatory variable was sarcopenia defined as muscle CSA at T10 two standard deviations below the mean of a healthy reference group reported by Derstine et al.^9^ We also explored associations with continuous muscle CSA (in 10 cm² steps) at T8, T10, and T12.^13^, ^15^, ^18^

### 2.4 Statistical analysis

Patient characteristics are presented using descriptive statistics including mean and standard deviation, or median and interquartile range. Differences in patient characteristics (sarcopenic vs. non‐sarcopenic; MGH vs. HDB) were assessed using t‐tests, χ², and Mann–Whitney *U*‐tests. Correlations between muscle measurements at different levels were assessed with Pearson correlations. Univariable relationships between sarcopenia, cancer‐specific survival, and overall survival were estimated using the Kaplan–Meier method and assessed for statistical significance with the log‐rank test. For multivariable analyses, Cox proportional hazard models were developed using a priori defined variables previously associated with survival following pneumonectomy for lung cancer: Age,^5^ body mass index (BMI),^5^ comorbidities (summarized using the Charlson Comorbidity Index [CCI], as previously validated in lung cancer resections),^2^, ^19^ forced expiratory volume in 1 second in % predicted (FEV1% pred),^5^ induction therapy,^5^ sex,^5^ smoking status,^20^ tumor stage,^5^ and side of pneumonectomy.^21^ Institution (MGH or HDB) was added as a covariable. Analyses were performed using sarcopenia (primary) and muscle at each level as a continuous variable (secondary).

The likelihood‐ratio test was used to assess whether the addition of sarcopenia or muscle improved model fit comparing the full models to restricted models without sarcopenia or muscle. Inter‐software and inter‐analyst agreement were assessed using intraclass correlation coefficients.^14^, ^16^ All analyses were performed using STATA (version 13.0, StataCorp). A type‐1 error rate of 5% was assumed for all confidence intervals and hypothesis tests.

## 3 RESULTS

### 3.1 Patient characteristics

Overall, 367 of 443 consecutive screened patients (82.8%) were included. Patient, tumor, and treatment details are summarized in Table 1.

**TABLE 1:** Patient characteristics, stratified by presence of sarcopenia. Median and interquartile range, mean and standard deviation or number, and percentage are given, as appropriate

	All (*n* = 367)	Sarcopenic (*n* = 104)	Non‐sarcopenic (*n* = 263)	*p* value
General patient characteristics				
Age, years, median (range)	62.2 (56.0–68.8)	64 (58.9–71.6)	60.7 (54.2–67.4)	**<0.001** ^a^
Body mass index, kg/m², median (range)	25.4 (22.9–28.5)	24.4 (22.2–26.3)	26.3 (23.1–29.6)	**<0.001** ^a^
Height, cm, mean (standard deviation)	171.4 (8.9)	172 (167.3–177.9)	171 (165–177)	0.29^c^
Weight, kg, median (range)	75 (65.4–87)	71 (65–80.2)	76 (66–90)	**0.003** ^a^
Male, n (%)	247 (67.3)	86 (82.7)	161 (61.2)	**<0.001** ^b^
FEV1% pred^d^, %, mean (standard deviation)	77.5 (19.2)	73.6 (18.3)	79.1 (19.4)	**0.014** ^c^
Patient comorbidities				
Charlson Comorbidity Index, median (range)	4 (3–5)	4 (4–5.5)	4 (3–5)	**0.001** ^a^
Prior myocardial infarction, *n* (%)	31 (8.4)	7 (6.7)	24 (9.1)	0.46^b^
Congestive heart failure, *n* (%)	0 (0)	0 (0)	0 (0)	1^b^
Chronic obstructive pulmonary disease, *n* (%)	69 (18.8)	21 (20.2)	48 (18.3)	0.67^b^
Diabetes mellitus, *n* (%)	24 (6.5)	8 (7.7)	16 (6.1)	0.57^b^
Smoking status, *n* (%)				0.09^b^
Never	36 (9.8)	6 (5.8)	30 (11.4)	
Former	222 (60.5)	60 (57.7)	162 (61.6)	
Current	109 (29.7)	38 (36.5)	71 (27.0)	
Tumor characteristics & treatment details				
Stage, *n* (%)				0.76^b^
I	13 (3.5)	5 (4.8)	8 (3.0)	
II	51 (13.9)	14 (13.4)	37 (14.1)	
IIIA	167 (45.5)	52 (50.0)	115 (43.7)	
IIIB	111 (30.3)	26 (25.0)	85 (32.3)	
IVA	18 (4.9)	5 (4.8)	13 (4.9)	
Tx	7 (1.9)	2 (1.9)	5 (1.9)	
Histology, *n* (%)				0.1^b^
Squamous cell	212 (57.8)	70 (67.3)	142 (54.0)	
Adenocarcinoma	120 (32.7)	29 (27.9)	91 (34.6)	
Large cell carcinoma	9 (2.5)	0 (0)	9 (3.4)	
Carcinoid	8 (2.2)	0 (0)	5 (1.9)	
Adenosquamous carcinoma	5 (1.4)	3 (2.9)	5 (1.9)	
Neuroendocrine carcinoma	4 (1.1)	0 (0)	4 (1.5)	
Bronchial gland type carcinoma	3 (0.8)	0 (0)	3 (1.1)	
Non‐small cell lung cancer, not otherwise specified	6 (1.6)	2 (1.9)	4 (1.5)	
Type of pneumonectomy, *n* (%)				0.93^b^
Standard pneumonectomy	329 (89.6)	93 (89.4)	236 (89.7)	
Carinal pneumonectomy	38 (10.4)	11 (10.6)	27 (10.3)	
Side of pneumonectomy, *n* (%)				0.85^b^
Right	163 (44.4)	47 (45.2)	116 (44.1)	
Left	204 (55.6)	57 (54.8)	147 (55.9)	
Induction therapy, *n* (%)	38 (10.4)	8 (7.7)	30 (11.4)	0.29^b^

Significance of bold means *p* value < 0.05.

^a^ Mann–Whitney *U* test.

^b^ *χ*² test.

^c^ *t* test.

^d^ FEV1% pred, forced expiratory volume in 1 second, in % predicted.

*p*‐values <0.05 are printed in bold numerics.

During the study period, the pneumonectomy rate compared to all resections for lung cancer was 3.8% at MGH and 11.3% at HDB. Differences in patient selection between the two institutions are reflected in a greater proportion of induction therapy and higher BMI at MGH, while HDB had a greater frequency of stage III disease and active smokers (Table S1). The indication for pneumonectomy in tumors pathologically staged as Tx, stage I or stage IVa is detailed in Table S2.

Mortality was 4.6% (17/367) at 30 days and 7.1% (26/367) at 90 days, without significant differences between the institutions. After a median follow‐up of 20.5 months, 183 of 367 patients (49.9%) had died. Of these deaths, 133 (72.7%) were classified as tumor‐specific, while 30 patients (16.4%) died of non‐cancer‐related causes (Table S3). The cause was unknown in 20 of 183 deaths (10.9%). Long‐term survival was similar between the institutions. Post‐surgical complications during the hospital stay are detailed in Table S4.

### 3.2 Muscle measurements

Criteria for sarcopenia were met by 104 of 367 (28.3%) patients. Patients with sarcopenia were more likely to be male, older, and have a lower BMI, lower preoperative FEV1, and more comorbidities (Table 1). The median muscle area was highest at T8 and lowest at T10, both with and without stratifying by sex (Table S5). The median muscle area was more than 50% higher in males compared to females (*p *< 0.001; Figure 2).

Compared to T10, our reference for study inclusion, muscle measurements were less frequently available at T8 (98.1%) compared to T12 (99.2%) due to incomplete visualization with a small field of view in seven patients at T8 and in three patients at T12. Muscle at all levels demonstrated moderate to strong correlation (Figure S1). Inter‐software and inter‐analyst agreement were excellent (Table S6).

### 3.3 Univariable analyses for sarcopenia as a binary explanatory variable

**FIGURE 3:**
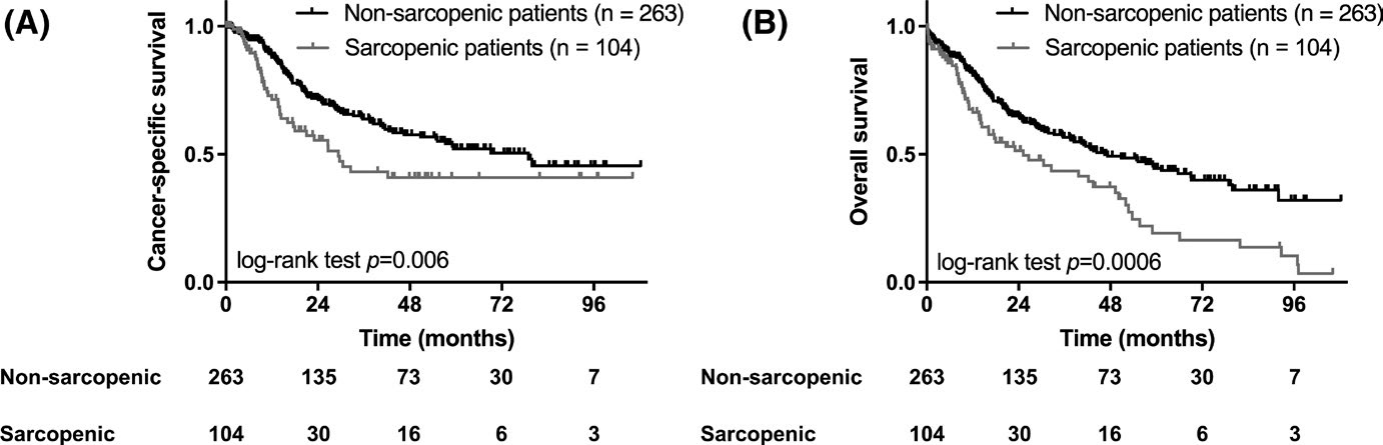
Kaplan–Meier plot illustrates cancer‐specific survival (A) and overall survival (B) following pneumonectomy for lung cancer in sarcopenic and non‐sarcopenic patients. Sarcopenic patients experienced significantly shorter cancer‐specific and overall survival when compared to non‐sarcopenic patients (*p *= 0.006 for CSS, *p *= 0.0006 for OS)

In univariable analyses, survival was shorter in sarcopenic patients compared to non‐sarcopenic patients (Figure 3). Median CSS was 29.4 months in sarcopenic and 78.9 months in non‐sarcopenic patients (*p *= 0.006). Median OS was 17.7 months in sarcopenic and 46.5 months in non‐sarcopenic patients (*p *= 0.0006).

### 3.4 Multivariable analyses for sarcopenia as a binary explanatory variable

In multivariable Cox proportional hazard regression models adjusted for parameters known to be associated with survival, sarcopenia was independently associated with cancer‐specific survival (hazard ratio [HR] 1.74, *p *= 0.008) and overall survival (HR 1.68, *p *= 0.003; Table 2 for CSS, Table S7 for OS).^2^, ^5^, ^13^, ^19^, ^20^, ^21^, ^22^

**TABLE 2:** Multivariable Cox proportional hazard regression of cancer‐specific survival after pneumonectomy for lung cancer. Disease stages are categorized as early (stages I, II), locally advanced (stage IIIA), and advanced (stage IIIB, IV)

	Sarcopenia (yes vs. no [reference]) *n* = 367		T8 (10 cm^2^) *n* = 360		T10 (10 cm^2^) *n* = 367		T12 (10 cm^2^) *n* = 364	
	HR (CI)	*p*	HR (CI)	*p*	HR (CI)	*p*	HR (CI)	*p*
Muscle	1.74 (1.16–2.60)	**0.008**	0.82 (0.73–0.91)	**<0.001**	0.81 (0.70–0.93)	**0.003**	1.00 (0.87–1.15)	0.96
Age, years	0.98 (0.95–1.01)	0.22	0.98 (0.95–1.01)	0.11	0.98 (0.95–1.01)	0.14	0.98 (0.96–1.01)	0.32
Body mass index, kg/m^2^	0.94 (0.90–0.99)	**0.023**	0.95 (0.90–0.99)	**0.029**	0.96 (0.91–1.02)	0.17	0.93 (0.87–0.99)	**0.024**
Charlson Comorbidity Index	1.24 (1.01–1.53)	**0.041**	1.21 (0.99–1.50)	0.07	1.25 (1.02–1.54)	**0.034**	1.25 (1.01–1.54)	**0.040**
FEV1% pred^a^, %	1.00 (0.99–1.01)	0.48	1.00 (1.00–1.01)	0.34	1.00 (0.99–1.01)	0.50	1.00 (0.99–1.01)	0.37
Induction therapy	1.56 (0.83–2.92)	0.16	1.57 (0.83–2.98)	0.17	1.53 (0.82–2.88)	0.18	1.56 (0.83–2.93)	0.17
Sex								
Male	Ref.		Ref.		Ref.		Ref.	
Female	0.75 (0.50–1.12)	0.16	0.27 (0.15–0.50)	**<0.001**	0.34 (0.19–0.61)	**<0.001**	0.66 (0.35–1.22)	0.18
Smoking status								
Never	0.99 (0.57–1.73)	0.98	0.95 (0.55–1.66)	0.87	0.99 (0.57–1.72)	0.98	0.93 (0.53–1.64)	0.81
Former	Ref.		Ref.		Ref.		Ref.	
Current	0.63 (0.41–0.97)	**0.036**	0.62 (0.40–0.97)	**0.034**	0.63 (0.41–0.98)	**0.039**	0.65 (0.42–1.01)	0.06
Stage								
I & II	0.34 (0.57–1.73)	**0.004**	0.34 (0.16–0.72)	**0.005**	0.37 (0.17–0.76)	**0.007**	0.38 (0.18–0.80)	**0.010**
Tx	0.70 (0.18–2.68)	0.60	0.56 (0.15–2.14)	0.39	0.63 (0.16–2.40)	0.50	0.79 (0.21–3.00)	0.72
IIIA	Ref.		Ref.		Ref.		Ref.	
IIIB & IV	1.55 (1.07–2.25)	**0.021**	1.49 (1.03–2.16)	**0.036**	1.55 (1.07–2.25)	**0.021**	1.57 (1.08–2.28)	**0.019**
Side of pneumonectomy								
Right	Ref.		Ref.		Ref.		Ref.	
Left	0.90 (0.63–1.28)	0.56	0.89 (0.62–1.27)	0.52	0.86 (0.61–1.22)	0.40	0.87 (0.61–1.24)	0.46
Institution								
MGH^b^	Ref.		Ref.		Ref.		Ref.	
HDB^c^	1.12 (0.62–2.03)	0.70	1.03 (0.57–1.88)	0.91	1.06 (0.59–1.91)	0.85	1.09 (0.61–1.97)	0.77
**Likelihood‐ratio test**								
Restricting muscle		**0.0093**		**0.0002**		**0.0031**		0.96

Significance of bold means *p* value < 0.05.

^a^ FEV1% pred, forced expiratory volume in 1 second, in % predicted.

^b^ MGH, Massachusetts General Hospital.

^c^ HDB, Heidelberg University Thoraxklinik.

*p*‐values <0.05 are printed in bold numerics.

### 3.5 Predictive capability

Likelihood ratio tests were performed to compare cancer‐specific and overall survival in models with and without muscle measurements, using sarcopenia as a categorical variable or muscle area as a continuous variable for each level, T8, T10, and T12, separately. Models that included muscle measurements demonstrated a significantly better fit than models excluding muscle (Table 2). The signal was strongest at T8, less strong at T10, while muscle measurements at T12 were not associated with either cancer‐specific or overall survival. These findings indicate that muscle area at T8 and T10, when combined with known predictors of survival, improves the prediction of cancer‐specific and overall survival following pneumonectomy in patients with NSCLC.

## 4 DISCUSSION

The present study analyzed surgical lung cancer cohorts from two tertiary care centers in North America and Europe to examine whether sarcopenia on routine preoperative chest CT predicts survival following pneumonectomy for NSCLC. Patients with sarcopenia based on the assessment of muscle mass at the level of the tenth thoracic vertebral body experienced significantly worse cancer‐specific and overall survival following pneumonectomy. Both sarcopenia and muscle mass at levels T8 and T10 were independently associated with cancer‐specific and overall survival in univariable and multivariable models. The likelihood‐ratio test confirmed that the addition of thoracic muscle mass increases the predictive capability of established risk prediction models. These findings strongly suggest that muscle on chest CT scans obtained as part of preoperative evaluation can be harnessed to improve the prediction of death from lung cancer following pneumonectomy.

The current study is the first to demonstrate a link between cancer‐specific survival and thoracic muscle mass. We prefer muscle assessment on preoperative chest CT scans to other radiographic examinations and bioimpedance because segmentation readily quantifies regional truncal muscle mass relevant to respiration and expectoration; guidelines already recommend chest CT, thereby avoiding additional tests and radiation exposure.^11^, ^14^, ^23^

Our findings expand the evidence relating muscle mass to cancer‐specific survival. The muscle area on abdominal CT at the level of the third lumbar vertebral body is an established predictor of cancer‐specific survival following resection of infra‐diaphragmatic malignancies.^24^, ^25^, ^26^, ^27^, ^28^ Psoas muscle area on preoperative abdominal CT has also been linked to OS in patients with lung cancer in a single‐center study.^12^ Our own single‐center study provided preliminary data suggesting that muscle area on chest CT at the level of T8 was also associated with OS following pneumonectomy but relied on a cohort‐specific cutoff value.^13^ A bi‐institutional study investigating overall mortality following various anatomic and non‐anatomic resections for lung cancer failed to uncover direct correlations between muscle area and survival and relied on a “morphometric index” with a cohort‐specific cutoff value.^29^ The investigators included anatomic lung resections equal or greater than segmentectomy and we suspect their pneumonectomy rate was low. Without reference to healthy controls, none of these studies firmly establish sarcopenia as a risk factor. The present study defines sarcopenia on chest CT through comparison with an independent cohort of healthy kidney donors, thereby avoiding the unreliability of cohort‐specific reference values.^9^

We included patients from two institutions in North America and Europe to achieve diversity in patient selection, radiographic technique, muscle assessment software, operative technique, and postoperative clinical management. This study, therefore, tested the association between muscle area and survival under various conditions and for the most extensive type of lung resection with the greatest perioperative risk. We excluded resections extending beyond the parietal pleura as well as completion pneumonectomies and pneumonectomy with a palliative intent given the different risk profiles of these resections.^30^, ^31^ Variations in patient selection criteria between the institutions imply that conclusions from this study may be widely applicable.

Our comprehensive pre‐specified model considers all established risk factors for mortality following pneumonectomy in patients with lung cancer, as well as the side of pneumonectomy^21^ and an institutional variable. We included the CCI to account for comorbidities as previously validated.^2^, ^19^ As expected, we found that higher tumor stage^5^ and male sex^5^ were negatively associated with survival. Smoking status,^20^ comorbidities,^2^ and induction therapy^32^—which requires careful patient selection in the neoadjuvant setting^33^—showed trends toward worse survival. Similar to prior studies, pulmonary function tests showed no significant effect.^13^ As previously demonstrated, age was not associated with survival, highlighting that mortality risk is not so much defined by chronologic, but morphologic age.^16^, ^34^, ^35^, ^36^

The choice of analyzed thoracic vertebral levels in this study was informed by prior reports and our own institutional experience.^13^, ^16^, ^23^ While we observed strong inter‐level correlations, muscle mass at the T8 level was the best predictor of survival, while muscle mass at T10 was inferior to T8 but still significant. We were surprised to find muscle mass at T12 showed no association with survival while muscle mass at T12 is most closely correlated with muscle mass at the commonly referenced L3 level.^9^ We suspect that shoulder muscles supporting upper extremity function which are visualized at T8 and to a lesser extent at T10, contribute to the ability of muscle mass at these levels to predict survival.^23^ We thus hypothesize that the combination of respiratory and shoulder musculature may be more helpful in survival prediction following pneumonectomy in patients with lung cancer than respiratory muscles alone.

There are some notable limitations to this study. First, the retrospective design precluded preoperative assessment of muscle strength and function (which some studies recommend^37^) and resulted in some exclusions due to missing data. However, the exclusion rate is low compared to other lung cancer body composition studies and we believe that our sample is representative.^13^, ^14^ Second, we had to retrospectively convert some TNM stages from the seventh to the eighth edition to achieve uniformity. Third, we chose to rely on pathologic instead of clinical staging given that pathologic staging was universally available and is strongly correlated with clinical stage.^38^ Last, we were unable to include race and ethnicity despite evidence that it affects body composition^39^ and outcomes in patients with lung cancer^40^ because these parameters are not commonly collected in Germany.

In conclusion, this transatlantic bi‐institutional study confirms sarcopenia on preoperative chest CT as an independent binary risk factor for both cancer‐specific and overall survival following pneumonectomy for lung cancer. Muscle mass as a continuous variable at vertebral levels T8 and T10 is also independently associated with survival following pneumonectomy for lung cancer. In the future, muscle mass on chest CT scans obtained as part of guideline‐concordant preoperative evaluation could be harnessed to improve mortality risk prediction in patients undergoing pneumonectomy for lung cancer.

## CONFLICTS OF INTEREST

F.J.F. was supported by the American Roentgen Ray Society Scholarship during the study period and has a related patent pending. T.M. and C.P.H. hold patents not related to this study. The other authors do not report relevant conflicts of interest.

## AUTHOR CONTRIBUTIONS

Fabian M. Troschel: Data curation, Formal analysis, Investigation, Methodology, Visualization, Writing ‐ original draft. Qianna Jin: Data curation, Investigation, Methodology, Writing ‐ review & editing. Florian Eichhorn: Investigation, Methodology, Writing ‐ review & editing. Thomas Muley: Data curation, Investigation, Methodology, Writing ‐ review & editing. Till D. Best: Data curation, Investigation, Validation, Writing ‐ review & editing. Konstantin S. Leppelmann: Data curation, Investigation, Writing ‐ review & editing. Amelie S. Troschel: Data curation, Investigation, Validation, Writing ‐ review & editing. Hauke Winter: Investigation, Supervision, Writing ‐ review & editing. Claus P. Heußel: Conceptualization, Investigation, Supervision, Writing ‐ review & editing. Henning A. Gaissert: Investigation, Methodology, Supervision, Writing ‐ review & editing. Florian J. Fintelmann: Conceptualization, Data curation, Investigation, Methodology, Project administration, Supervision, Writing ‐ review & editing.

## Supporting information

Supplementary Material

## Data Availability

Research data (including CT scans) cannot be shared for ethical reasons given the scarcity of the investigated procedure and, thus, the identifiability of individual patients.
