# Supplementary material for: Sarcopenia on preoperative chest computed tomography predicts cancer‐specific and all‐cause mortality following pneumonectomy for lung cancer: A multicenter analysis
Source: Cancer Med. 2021 Aug 19;10(19):6677–86. doi: 10.1002/cam4.4207 (PMC8495285; doi:10.1002/cam4.4207)
Supplement: Supplementary file 1 — Supplementary Material [file CAM4-10-6677-s001.docx]

**SUPPLEMENTARY MATERIAL**

**Supplementary Table 1**: **Patient characteristics, stratified by institution**. Median and interquartile range, mean and standard deviation or number and percentage are given, as appropriate.

|  | All (n=367) | Massachusetts General Hospital (n=72) | Heidelberg University Thoraxklinik (n=295) | *p* value |
| --- | --- | --- | --- | --- |
| **General patient characteristics** | | | | |
| Age, years, median (range) | 62.2 (56.0-68.8) | 59.5 (53.0-70.0) | 62.4 (56.7-68.6) | 0.08^a^ |
| Body mass index, kg/m², median (range) | 25.4 (22.9-28.5) | 27.0 (23.8-30.6) | 25.1 (22.8-28.3) | **0.006**^a^ |
| Height, cm, mean (standard deviation) | 171.4 (8.9) | 170.9 (9.6) | 171.5 (8.8) | 0.62^c^ |
| Weight, kg, median (range) | 75 (65.4-87) | 78.7 (68.5-90.0) | 75.0 (65.0-86.0) | 0.07^a^ |
| Male, n (%) | 247 (67.3) | 43 (59.7) | 204 (69.2) | 0.13^b^ |
| FEV1 % pred^d^, %, mean (standard deviation) | 77.5 (19.2) | 74.8 (16.7) | 78.2 (19.8) | 0.19^c^ |
| **Patient comorbidities** | | | | |
| Charlson Comorbidity Index, median (range) | 4 (3-5) | 4 (3-5) | 4 (3-5) | 0.10^a^ |
| Prior myocardial infarction, n (%) | 31 (8.4) | 4 (5.5) | 27 (9.2) | 0.33^b^ |
| Congestive heart failure, n (%) | 0 (0) | 0 (0) | 0 (0) | 1^b^ |
| Chronic obstructive pulmonary disease, n (%) | 69 (18.8) | 8 (11.1) | 61 (20.7) | 0.06^b^ |
| Diabetes mellitus, n (%) | 24 (6.5) | 5 (6.9) | 19 (6.4) | 0.88^b^ |
| Smoking status, n (%) |  |  |  | 0.054^b^ |
| Never | 36 (9.8) | 8 (11.1) | 28 (9.5) |  |
| Former | 222 (60.5) | 51 (70.8) | 171 (58.0) |  |
| Current | 109 (29.7) | 13 (18.1) | 96 (32.5) |  |
| **Tumor characteristics & treatment details** | | | | |
| Stage, n (%) |  |  |  | **<0.001**^b^ |
| I | 13 (3.5) | 6 (8.3) | 7 (2.4) |  |
| II | 51 (13.9) | 28 (38.9) | 23 (7.8) |  |
| IIIA | 167 (45.5) | 27 (37.5) | 140 (47.5) |  |
| IIIB | 111 (30.3) | 2 (2.8) | 109 (37.0) |  |
| IVA | 18 (4.9) | 2 (2.8) | 16 (5.4) |  |
| Tx | 7 (1.9) | 7 (9.7) | 0 (0) |  |
| Histology, n (%) |  |  |  | 0.08^b^ |
| Squamous cell | 212 (57.8) | 40 (55.6) | 172 (58.3) |  |
| Adenocarcinoma | 120 (32.7) | 25 (34.7) | 95 (32.2) |  |
| Large cell carcinoma | 9 (2.5) | 2 (2.8) | 7 (2.4) |  |
| Carcinoid | 8 (2.2) | 0 (0) | 8 (2.7) |  |
| Adenosquamous carcinoma | 5 (1.4) | 0 (0) | 5 (1.7) |  |
| Neuroendocrine carcinoma | 4 (1.1) | 1 (1.4) | 3 (1.0) |  |
| Bronchial gland type carcinoma | 3 (0.8) | 0 (0) | 3 (1.0) |  |
| Non-small cell lung cancer, not otherwise  specified | 6 (1.6) | 4 (5.6) | 2 (0.7) |  |
| Type of pneumonectomy, n (%) |  |  |  | 0.84^b^ |
| Standard pneumonectomy | 329 (89.6) | 65 (90.3) | 264 (89.5) |  |
| Carinal pneumonectomy | 38 (10.4) | 7 (9.7) | 31 (10.5) |  |
| Side of pneumonectomy, n (%) |  |  |  | 1^b^ |
| Right | 163 (44.4) | 32 (44.4) | 131 (44.4) |  |
| Left | 204 (55.6) | 40 (55.6) | 164 (55.6) |  |
| Induction therapy, n (%) | 38 (10.4) | 24 (33.3) | 14 (4.8) | **<0.001**^b^ |

^a^ Mann-Whitney U test; ^b^ χ² test; ^c^ t test, ^d^ FEV1 % pred, forced expiratory volume in 1 second, in % predicted

**Supplementary Table 2**: **Reasons for pneumonectomy in patients with tumors classified as Tx, stage I and stage IVa.**

| Stage I (n=13) | - Neoadjuvant treatment resulted in downstaging (n=4) - Inflammatory involvement of central structures requiring pneumonectomy (n=8) - Oncologic bilobectomy extended to pneumonectomy due to non-functional remaining lobe 2^nd^ to tuberculosis (n=1) |
| --- | --- |
| Stage IVa (n=18) | - Oligometastatic lung cancer in good surgical candidates (n=18) |
| Tx (n=7) | - Neoadjuvant treatment with complete response (n=6) - A primary was first treated with definitive chemoradiotherapy. When tumor spread to the contralateral lung was noted, a pneumonectomy was performed for this second manifestation (n=1) |

**Supplementary Table 3**: **Postoperative survival, stratified by institution**. Number and percentage are given.

|  | All (n=367) | Massachusetts General Hospital (n=72) | Heidelberg University Thoraxklinik (n=295) | p value |
| --- | --- | --- | --- | --- |
| **Short-term survival** | | | | |
| 30-day mortality | 17 (4.6) | 2 (2.8) | 15 (5.1) | 0.40^a^ |
| 90-day mortality | 26 (7.1) | 4 (5.6) | 22 (7.5) | 0.57^a^ |
| **Long-term survival** | | | | |
| Long-term overall survival | 183 (49.9) | 35 (48.6) | 148 (50.2) | 0.82^a^ |
| Long-term cancer-specific survival | 133 (36.2) | 20 (27.8) | 113 (38.3) | 0.10^a^ |

^a^χ² test

**Supplementary Table 4**: **Postoperative in-hospital complications**. Median and interquartile range or number and percentage are given, as appropriate.

|  | All (n=367) | Massachusetts General Hospital (n=72) | Heidelberg University Thoraxklinik (n=295) | p value |
| --- | --- | --- | --- | --- |
| Length of hospital stay (days) | 15 (12-20) | 6.5 (5-7) | 17 (14-21) | **<0.001**^a^ |
| Deceased during hospital stay, n (%) | 16 (4.4) | 2 (2.8) | 14 (4.8) | 0.46^b^ |
| Pneumonia, n (%) | 30 (8.2) | 7 (9.7) | 23 (7.8) | 0.59^b^ |
| Respiratory failure, n (%) | 16 (4.4) | 7 (9.7) | 9 (3.1) | **0.013**^b^ |
| Reintubation, n (%) | 15 (4.1) | 6 (8.3) | 9 (3.1) | **0.042**^b^ |
| Pulmonary embolism, n (%) | 2 (0.5) | 1 (1.4) | 1 (0.3) | 0.28^b^ |
| Sepsis, n (%) | 5 (1.4) | 0 (0) | 5 (1.7) | 0.27^b^ |
| Recurrent laryngeal nerve injury, n (%) | 9 (2.5) | 6 (8.3) | 3 (1.0) | **<0.001**^b^ |
| Delirium, n (%) | 7 (1.9) | 2 (2.8) | 5 (1.7) | 0.55^b^ |
| Renal Failure, n (%) | 7 (1.9) | 2 (2.8) | 5 (1.7) | 0.55^b^ |

^a^ Mann-Whitney U test; ^b^ χ² test

**Supplementary Table 5:** **Muscle cross-sectional area and incidence of sarcopenia, stratified by institution**. Measurements were performed at the level of the eighth (T8), tenth (T10) and twelfth (T12) thoracic vertebral body. Median and interquartile range or number and percentage are given, as appropriate.

|  | All (n=367) | Massachusetts General Hospital (n=72) | Heidelberg University Thoraxklinik (n=295) | p value |
| --- | --- | --- | --- | --- |
| **T8** (all; cm²) | 104.5 (79.7-123.9) | 105.0 (78.0-127.3) | 104.2 (79.3-123.2) | 0.95^a^ |
| T8 (male; cm²) | 116.9 (103.7-131.9) | 123.9 (107.0-138.3) | 116.3 (102.7-129.2) | 0.10^a^ |
| T8 (female; cm²) | 73.9 (69.3-81.0) | 75.9 (71.6-82.6) | 72.7 (68.8-80.8) | 0.34^a^ |
| **T10** (all; cm²) | 87.7 (68.2-105.3) | 88.7 (70.3-105.3) | 87.5 (67.4-105.3) | 0.99^a^ |
| T10 (male; cm²) | 98.6 (86.5-111.0) | 101.5 (90.7-112.5) | 98.3 (86.1-110.5) | 0.20^a^ |
| T10 (female; cm²) | 63.9 (58.0-70.7) | 66.0 (59.1-71.4) | 63.3 (57.9-69.5) | 0.40^a^ |
| **T12** (all; cm²) | 101.8 (78.3-118.1) | 104.6 (80.8-116.4) | 101.4 (77.3-118.8) | 0.81^a^ |
| T12 (male; cm²) | 112.3 (99.9-125.1) | 114.0 (105.4-128.3) | 112.1 (98.0-125.1) | 0.19^a^ |
| T12 (female; cm²) | 72.2 (63.8-81.2) | 77.6 (67.0-85.3) | 70.5 (63.5-77.4) | **0.04**^a^ |
| **Sarcopenia at T10** (n (%)) | 104 (28.3) | 18 (25.0) | 86 (29.2) | 0.48^b^ |

^a^ Mann-Whitney U test; ^b^ χ² test

**Supplementary Table 6**: **Intraclass correlation coefficients for muscle measurements with confidence intervals.**

|  | T8  eighth thoracic vertebra | T10  tenth thoracic vertebra | T12  tenth thoracic vertebra |
| --- | --- | --- | --- |
| Inter-analyst comparison at Massachusetts General Hospital | 0.984 (0.960-0.993) | 0.985 (0.966-0.993) | 0.985 (0.966-0.993) |
| Inter-analyst comparison at Heidelberg University Thoraxklinik | 0.990 (0.975-0.996) | 0.978 (0.978-0.991) | 0.983 (0.960-0.993) |
| Inter-software comparison between Massachusetts General Hospital and Heidelberg University Thoraxklinik | 0.991 (0.979-0.996) | 0.986 (0.969-0.994) | 0.976 (0.944-0.990) |


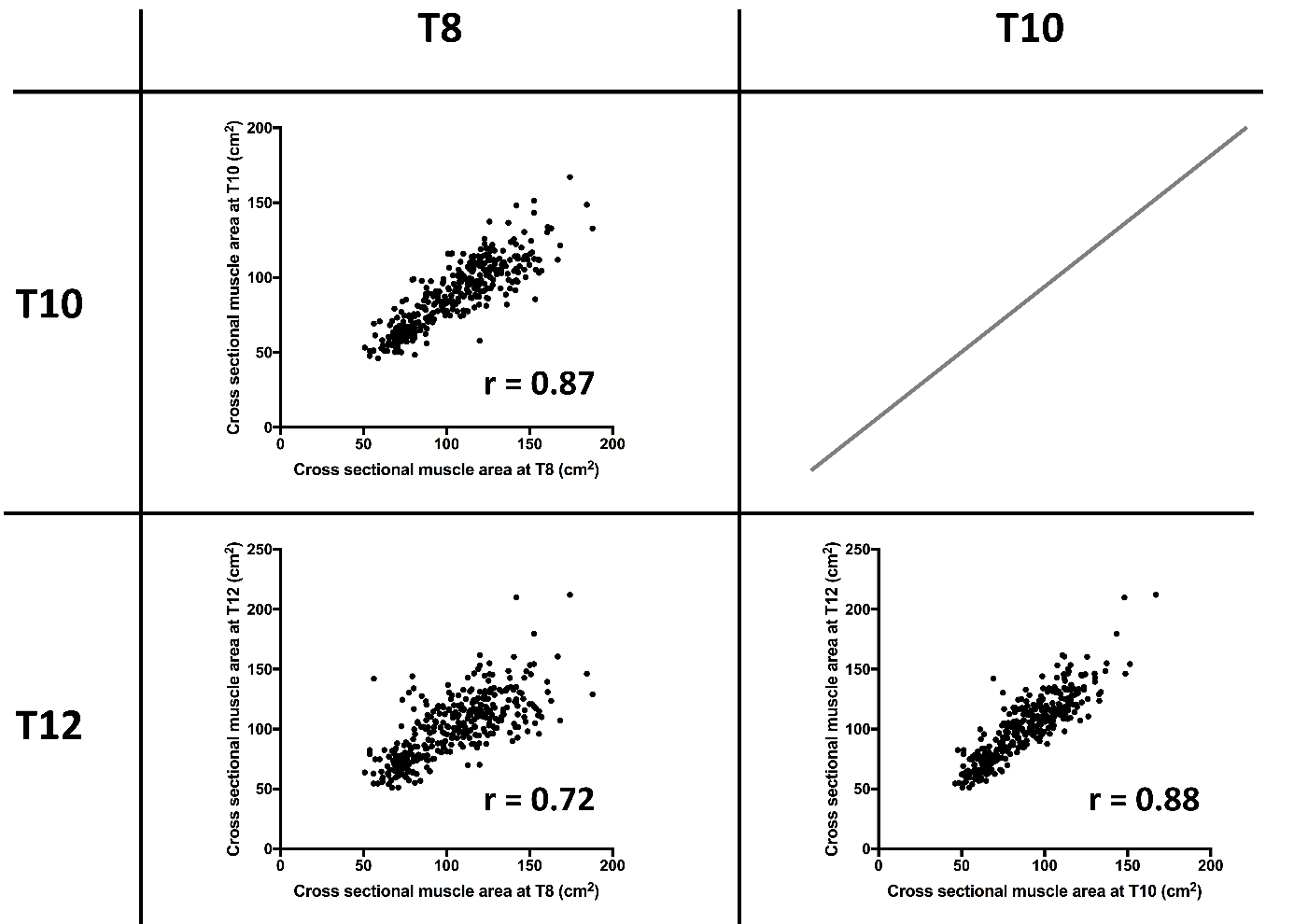


**Supplementary Figure 1**: **Correlations between muscle cross-sectional area at the level of the eighth (T8), tenth (T10) and twelfth (T12) thoracic vertebral bodies**. Pearson’s r values indicate strong correlations.

**Supplementary Table 7:** **Multivariable Cox proportional hazard regression of overall survival after pneumonectomy for lung cancer.** Disease stages are categorized as early (stage I, II), locally advanced (stage IIIA) and advanced (stage IIIB, IVa).

|  | Sarcopenia  (yes vs. no [reference])  n = 367 | | T8 (10cm^2^)  n = 360 | | T10 (10cm^2^)  n = 367 | | T12 (10cm^2^)  n = 364 | |
| --- | --- | --- | --- | --- | --- | --- | --- | --- |
|  | HR (CI) | p | HR (CI) | p | HR (CI) | p | HR (CI) | p |
| Muscle | 1.68 (1.19-2.38) | **0.003** | 0.85 (0.78-0.93) | **0.001** | 0.84 (0.75-0.94) | **0.004** | 0.99 (0.88-1.12) | 0.90 |
| Age, years | 1.00 (0.97-1.02) | 0.87 | 0.99 (0.97-1.02) | 0.62 | 0.99 (0.97-1.02) | 0.69 | 1.00 (0.98-1.03) | 0.94 |
| Body mass index, kg/m^2^ | 0.96 (0.92-1.00) | 0.073 | 0.96 (0.92-1.00) | 0.07 | 0.98 (0.93-1.02) | 0.32 | 0.95 (0.90-1.00) | 0.056 |
| Charlson Comorbidity Index | 1.16 (0.97-1.39) | 0.11 | 1.14 (0.95-1.36) | 0.15 | 1.16 (0.97-1.39) | 0.10 | 1.16 (0.97-1.39) | 0.10 |
| FEV1 % pred^a^, % | 1.00 (0.99-1.01) | 0.71 | 1.00 (0.99-1.01) | 0.55 | 1.00 (0.99-1.01) | 0.75 | 1.00 (0.99-1.01) | 0.66 |
| Induction therapy | 1.50 (0.89-2.53) | 0.13 | 1.52 (0.90-2.59) | 0.12 | 1.48 (0.88-2.50) | 0.14 | 1.48 (0.88-2.49) | 0.14 |
| Sex |  |  |  |  |  |  |  |  |
| Male | Ref. |  | Ref. |  | Ref. |  | Ref. |  |
| Female | 0.83 (0.59-1.17) | 0.28 | 0.36 (0.22-0.60) | **<0.001** | 0.42 (0.26-0.69) | **0.001** | 0.71 (0.43-1.20) | 0.20 |
| Smoking status |  |  |  |  |  |  |  |  |
| Never | 0.81 (0.48-1.37) | 0.42 | 0.78 (0.46-1.32) | 0.36 | 0.81 (0.48-1.36) | 0.42 | 0.76 (0.44-1.29) | 0.30 |
| Former | Ref. |  | Ref. |  | Ref. |  | Ref. |  |
| Current | 0.87 (0.62-1.23) | 0.44 | 0.86 (0.61-1.22) | 0.41 | 0.87 (0.48-1.36) | 0.45 | 0.89 (0.63-1.27) | 0.53 |
| Stage |  |  |  |  |  |  |  |  |
| I & II | 0.40 (0.23-0.69) | **0.001** | 0.40 (0.23-0.70) | **0.001** | 0.42 (0.24-0.73) | **0.002** | 0.43 (0.25-0.75) | **0.003** |
| Tx | 0.61 (0.19-1.89) | 0.39 | 0.51 (0.16-1.59) | 0.25 | 0.55 (0.17-1.71) | 0.30 | 0.66 (0.21-2.08) | 0.48 |
| IIIA | Ref. |  | Ref. |  | Ref. |  | Ref. |  |
| IIIB & IV | 1.25 (0.91-1.72) | 0.18 | 1.22 (0.88-1.68) | 0.23 | 1.24 (0.90-1.71) | 0.19 | 1.23 (0.89-1.70) | 0.201 |
| Side of pneumonectomy |  |  |  |  |  |  |  |  |
| Right | Ref. |  | Ref. |  | Ref. |  | Ref. |  |
| Left | 0.78 (0.58-1.05) | 0.10 | 0.77 (0.57-1.03) | 0.08 | 0.75 (0.56-1.01) | 0.06 | 0.77 (0.57-1.03) | 0.08 |
| Institution |  |  |  |  |  |  |  |  |
| MGH^b^ | Ref. |  | Ref. |  | Ref. |  | Ref. |  |
| HDB^c^ | 0.84 (0.53-1.33) | 0.45 | 0.80 (0.50-1.28) | 0.36 | 0.80 (0.50-1.27) | 0.34 | 0.83 (0.53-1.31) | 0.43 |
| **Likelihood-ratio test** | | | | | | | | |
| Restricting muscle |  | **0.0005** |  | **0.0039** |  | **0.0036** |  | 0.90 |

^a^ FEV1 % pred, forced expiratory volume in 1 second, in % predicted. ^b^ Massachusetts General Hospital; ^c^ Heidelberg University Thoraxklinik.
